# Towards leaving no one behind in North Macedonia: a mixed methods assessment of barriers to effective coverage with health services

**DOI:** 10.1186/s12939-023-02082-3

**Published:** 2024-03-15

**Authors:** Theadora Swift Koller, Jelena Kjetkovikj Janeva, Elena Ognenovska, Ana Vasilevska, Simona Atanasova, Chris Brown, Antoni Dedeu, Anne Johansen

**Affiliations:** 1 Department for Gender Equality, Human Rights and Health Equity, WHO Headquarters, Geneva, Switzerland; 2 TIM Institute, Skopje, North Macedonia; 3 World Health Organization Country Office in North Macedonia, Skopje, North Macedonia; 4 WHO European Office for Investment for Health and Development, Venice, Italy; 5 WHO European Centre for Primary Health Care, Almaty, Kazakhstan

**Keywords:** Barriers, Health services, Health equity, Primary health care, Health systems, Universal health coverage, Social determinants, Gender, Health workforce, Health financing

## Abstract

**Background:**

The Government of North Macedonia’s Primary Health Care reform is committed to leaving no one behind on the path to Universal health Coverage (UHC). During mid-2022 to March 2023, the World Health Organization (WHO) collaborated with the Government and other national stakeholders for an assessment of barriers to effective coverage with health services experienced by adult citizens, with a specific focus on rural areas and subpopulations in situations of vulnerability.

**Methods:**

This study constituted the piloting of a draft forthcoming WHO handbook on assessing barriers for health services, grounded in the Tanahashi framework for effective coverage with health services. In North Macedonia, the convergent parallel mixed methods study involved four sources. These were: a nationally representative Computer Assisted Telephone Interview Survey (1,139 respondents); 24 key informant interviews with representatives from government, professional associations, non-governmental and civil society organizations, and development partners; 12 focus groups in four regions with adults from vulnerable/high risk groups in rural areas and small urban settlements and an additional focus group with persons with disabilities; and a literature review. Instrument design was underpinned by the Tanahashi framework, which also orientated data triangulation and deductive analysis. The research team synergistically incorporated emerging themes in an inductive way. A key component of the assessment was participatory design of the study protocol with inputs from national stakeholders as well as participatory deliberation of the results and the ways forward.

**Results:**

Despite considerable progress towards UHC in North Macedonia, the assessment elucidated remaining challenges. These included: insufficient numbers of health workers, in general and particularly in the more disadvantaged regions of the country; inadequate number of outpatient medicines covered by health insurance; distance and transportation obstacles, including indirect travel costs, particularly in rural areas; adverse gender norms and relations for both women and men inhibiting timely treatment seeking; perceived discrimination by providers on multiple grounds; bottlenecks including waiting times to get appointments for specialist referrals; and lack of patient adherence, due several factors including costs of medicines and health products.

**Conclusions:**

The outputs from this study of barriers to effective coverage with health services for adult citizens of North Macedonia are feeding into the ongoing Primary Health Care reform, and provide evidence for equity-related actions in the forthcoming National Development Strategy.

## Background and study objectives

Life expectancy in North Macedonia has improved over the past few decades and important advancements have been made in progress towards Universal Health Coverage [1, 2]. Challenges remain, however, as outlined in the National Health Strategy 2021–2030, regarding both service coverage and financial protection [3]. Unequal access to affordable, high-quality health care services and inadequate financial protection resulting in high out-of-pocket expenditures are persisting drivers of health inequities in North Macedonia [4].

To address these challenges, and despite the disruptions caused by COVID-19, important advances are being made through the Primary Health Care (PHC) reform. The orientations for the reform are delineated in the *National strategy and action plan for implementing the primary health care reform *[5]. The strategy and action plan include a focus on enhanced competencies of primary care doctors and nurses; monitoring of PHC; strengthening home-based care and outreach capacity; reorienting multi-profile primary care teams and models of care towards better addressing noncommunicable diseases and maternal, newborn and child health; and integrated social and health sector services for older people and other people with complex chronic conditions [5].

The evidence that has informed the reform to date has largely looked at health system performance issues at an aggregate level, as documented in the WHO Regional Office for Europe 2019 report “Primary health care organization, performance and quality in North Macedonia” [6]. Lesser attention has been given to inequities in experiences by individuals of health service usage or unmet need, exploring differences by sex and age, across the social gradient (by income, education, etc.), ethnicities, and spatial (i.e., rural, urban, regional) dimensions.

In support of advancing health equity through the PHC reform (and linked reforms in areas such as health financing), a mixed methods assessment was conducted to contribute to understanding the supply- and demand-side barriers to effective health services coverage. The assessment focused on barriers at the interface between the services and the [prospective or actual] patient. The study aimed to deepen understanding of why some subpopulations are being left behind, hence providing evidence for reducing health inequities, closing coverage gaps, acting on social and environmental determinants, and – in general – contributing to PHC-oriented health system strengthening.

The study objectives were: (1) to explore the barriers and facilitating factors to effective health service coverage in North Macedonia, with an explicit but not exclusive focus on rural populations and people in small urban settlements and with particular attention to age, sex and gender, disability status, income and other social factors that can influence experiences of vulnerability and deprivation; and (2) to highlight opportunities to improve health equity and equity in access to high-quality health services by strengthening the PHC-oriented health system and through cross-sectoral action on key determinants of health.

Contributing to filling the above-mentioned equity data gap for North Macedonia, this study has attempted to add value through its focus on areas and subpopulations experiencing exacerbated social and spatial disadvantage in relation to health and its determinants. In addition, the study provides insights to demand-side perspectives and the compounding and intersecting nature of barriers (i.e., how different barriers can combine to derail/obstruct or delay progression along a patient pathway).

## Methods

### Methods overview

The main research question underpinning the study was: “What barriers (and facilitating factors) to effective coverage with health services are experienced by adults in North Macedonia – in particular those living in rural areas and small urban settlements – and by subpopulations in vulnerable situations?”.

This advanced convergent parallel mixed methods study was informed by a *WHO draft handbook for conducting assessments of barriers to effective coverage with health services (forthcoming*) [7]. The handbook is grounded in the Tanahashi framework for effective coverage [8]. This framework can be used to explore barriers and facilitating factors in relation to service availability, accessibility, acceptability, contact and effective coverage, with effective coverage defined as when people in need of services receive these services of sufficient quality to obtain potential health gains [7, 9]. The Tanahashi framework underpins both the qualitative and quantitative dimensions of the study and data triangulation. All instruments were designed to cover the subdomains in the below Table 1, which draws from the WHO draft handbook. The Table reflects insights and lessons learnt from previous WHO barriers assessments and Innov8 [10] applications conducted over the past 10 years, as well as specific adaptations for the North Macedonian context.

**Table 1:** Guide for data analysis using the Tanahashi framework for effective coverage

*Barrier domain*	*Types of barriers that can be experienced across the continuum of health services, with the respective shorthand code for data processing*
**Availability** *[folder: availability]*	• Insufficient number or density of health facilities; **[code: facility]**
	• No outreach mechanisms/ community-based service points; **[code: outreach]**
	• Insufficient supply and appropriate stock of health workers, with the competencies (including through access to ongoing training), and skill‐mix to match the health needs of the population; **[code: HW mix and competencies]**;
	• Lack of equitable distribution of health workers taking into account the demographic composition, rural‐urban mix and under‐served areas or populations; **[code: HW distribution]**
	• Lack of medicines responding to population needs; **[code: medications availability]**
	• Scarcity or poor quality of, or insufficient maintenance of necessary equipment (e.g. equipment for exams, wheelchairs for patients, etc.); **[code: equipment]**
	• Weak laboratory system or inadequate cold chain; **[code: lab cold chain]**
	• Services not available in any location perceived as close enough to be realistically reachable, for the given health condition of concern [e.g., cancer or other NCDs services only available in capitol city or abroad, GBV services only available in capitol; **[code: no service]**
	• Inadequate ambulance services and/or transport methods/vehicles for mobile health units/home-care visits; **[code: no med vehicle]**
	• Shortage or poorly functioning basic amenities like electrification, improved water and sanitation, and waste management in health facilities; **[code: amenities]**
	• Lack of adequate computer equipment, IT connectivity and phone services (including for outreach services, telemedicine, by either provider or patient). **[code: ITC equipment]**
**Accessibility** *[folder: acessibility]*	*Geographic and physical:*
	- Distance and time for travelling to health service point; **[code: travel time]**
	- Lack of appropriate mode of transport; **[code: travel method]**
	- Unsafe terrain or weather conditions, impassable roads due to quality, road blockages due to conflict/insecurity, unsafe location of service point; **[code: unsafe conditions]**
	- physical accessibility of facilities for people with physical and/or cognitive disabilities; **[code: accessible for disabled]**
	*Financial – covering both financial barriers and drivers of financial hardship*
	• Direct: official out-of-pocket expenditures for services (e.g. co-payment for services, laboratory tests, exams); **[code: direct service costs]**
	• Direct: official out-of-pocket for medicines and health products (e.g., assistive devices); **[code: direct medprod costs]**
	• Indirect: transport and accommodation costs linked to using services; **[code: indirect transport costs]**
	• Indirect: opportunity costs (e.g. lost work, costs of child or elder care in absence, paying someone to do one’s job during absence (e.g., manage livestock/farm); **[code: indirect opportunity costs]**
	• Informal payments (cash or in-kind). **[code: informal payments]**
	• Public health service capacity and provider incentive structure influencing patients use of private services; **[code: private public interface]**
	*Organizational and informational:*
	• Attention to opening times in synergy with when people are available to access services; **[code: opening times]**
	• Systems to schedule appointments and waiting times/timeliness; **[code: scheduling]**
	• Administrative requirements for care (e.g. registration in local area); **[code: registration]**
	• Lack of access to culturally and linguistically appropriate health information, which consider some populations’ world views and cultural practices; **[code: culturally relevant info]**
	• Delivery of health information not considering the most appropriate communication modalities influenced by, for example illiteracy rates, limited access to technology and internet connectivity, preferred use of TV or radio over written materials, etc. **[code: communication modality]**
	• Lack of awareness of rights and obligations (demand-side)** [code: awareness]**
	*See acceptability for: Barriers related to power dynamics and inequalities (e.g., resulting in lack of autonomy to make decisions about one’s own health) and fear of/previous experiences of discrimination based on gender, ethnicity and other grounds that make people not want to access services*
**Acceptability** *[folder: acceptability]*	• Cultural beliefs and preferences (e.g. differing views of health and illness) and coordination/integration with indigenous/traditional medicine systems; **[code: culturally acceptable services]**
	• Gender norms, roles, power and relations which inhibit access (e.g. limited autonomy of some women in deciding when to seek care, patient only being allowed to or wanting to see a same sex provider, or gender norms on masculinity that delay treatment seeking); **[code: gender norms]**
	• Age-appropriateness of services (e.g. are adolescent-friendly services provided); **[code: age]**
	• Services that account for biological differences by sex (e.g., CVD services that account for specific differences in manifestation of symptoms of a heart attack between men and women); **[code: biological differences]**
	• Negative perceptions of service quality (including the quality dimensions of equity, safety, effectiveness, people-centredness, efficiency, timeliness, and integration); **[code: quality perceptions]**
	• Trust in the health system (linked to perceptions of transparency and accountability, and experience with corruption); **[code: trust]**
	• Discriminatory attitudes by providers (e.g. based on sex, gender, ethnicity, marital status, religion, caste, disability, health status, or sexual orientation of the person seeking care); **[code: discrimination]**
	• Extent to which confidentiality is protected; **[code: confidentiality]**
	• Attractiveness compared to alternative/competing options for using one’s time and resources (e.g. health promotion services are available, accessible and acceptable, but less attractive compared to other activities). **[code: prioritization]**
**Contact**	Contact coverage refers to the actual contact between the service provider and the user when services are available, accessible and acceptable
**Effective coverage ***[folder: effective coverage]*	• Lack of diagnostic accuracy (influenced by lack of diagnostic equipment and other factors such as gender unequal/blind protocols); **[code: diagnostic capacity]**
	• Insufficient provider compliance (e.g. related to low levels of training, lack of supportive system requirements such as protocols and guidelines, and deficient overall quality control mechanisms); **[code: provider compliance]**
	• Weak referral and back-referral systems; **[code: referrals]**
	• Inadequate treatment adherence (e.g. due to unclear instructions, poor patient-provider relationship, mismatch between treatment prescribed and patient compliance ability, adverse social conditions and gender roles/relations); **[code: patient adherence]**
	• Stigmatization caused by service usage that disrupts treatment adherence and/or otherwise negatively impacts patients’ health. **[code: stigmatization]**
	• Dual practice influencing patient pathways and service costs **[Code: dual practice]**
	• Lack of integrated care for people with comorbidities and/or social-health care for person requiring both synergistically **[Code: integrated care]**
	*Link to financial hardship dimension of UHC:* *Catastrophic or impoverishing expenditures or detrimental sale of assets incurred during the process of care that force the patient to stop treatment before it is completed hence impacting on effective coverage (and looping back to barriers under financial accessibility).* **[code: financial hardship]**

Source: The authors, drawing from the WHO draft handbook for conducting assessments of barriers to effective coverage with health services (forthcoming)

The need for the study was identified by WHO in keeping with priorities on social determinants of health and health equity, as well as on Primary Health Care, in the *WHO and Government of North Macedonia Biennial Collaborative Agreement for 2022–2023*. The Tim Institute (TИM Инcтитyт), a research institute in North Macedonia with experience in running both qualitative and quantitative studies across sectoral domains, was commissioned to execute the research, working closely with WHO. Across the study design and subsequent research process, ongoing guidance and technical inputs were provided through weekly meetings and co-design/co-authoring work by WHO staff (from Headquarters, Regional and Country Office levels). At key decision-making points, meetings were held with an oversight committee comprised of members of the Ministry of Health and Health Insurance Fund.

**Fig. 1:**
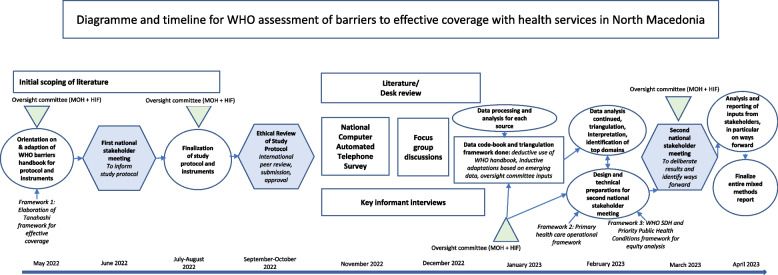
Diagramme and timeline for the WHO assessment of barriers to effective coverage with health services in North Macedonia

Figure 1 below provides an overview of all study components and the timeline for execution. This convergent parallel mixed methods study included primary data collection through a Computer Assisted Telephone Interview (CATI) survey, key informant interviews at national and subnational levels, and focus groups, done alongside a desk/literature review. Adding to the convergent parallel mixed methods approach to the assessment, the methods also included a participatory dimension. These are described in the sections that follow.

### Ethical clearance

The study received approval from the Ethics Committee for Human Research, from the Medical Faculty at the Ss. Cyril and Methodius University in Skopje, as well as the global WHO Research Ethics Review Committee. All participants in the study were asked for consent, in the appropriate language, and only those who provided consent were included. All research was carried out in keeping with the standards of the World Medical Association’s 1964 Declaration of Helsinki and the Council for International Organizations of Medical Sciences international ethical guidelines, as well as WHO’s ethical standards and procedures for research with human beings.

### Study location

The Republic of North Macedonia is a Member State of the WHO European Region, located in south-eastern Europe. According to the State Statistical Office, North Macedonia had a total enumerated population of 2,097,319 in 2022 [11]. The percentage of the population aged 65 years and over has been increasing, from 11.9% to 17.7% during the period 2012–2022 [12]. The ethnic affiliations of the population include Macedonian, Albanian, Turkish, Roma, Vlach, Serbian, Bosniaks and other/unknown, with Macedonian representing the majority of the population at 54.21%% followed by Albanian at 24.30% [11, 12]. In 2019, almost 4 in 10 people (40%) in North Macedonia were at risk of poverty or severe material deprivation or were living in households with very low work intensity [13]. Noncommunicable diseases account for about 95% of all deaths in the country [1].

The assessment was national in scope, with qualitative work done also in four subnational locations (East, Pelagonia, Polog and Southeast), selected on the basis of deprivation levels andother factors. The Human Development Index [14] was used as a statistic composite index of life expectancy, education and per capita income indicators to select the most disadvantaged regions. This was complemented by analysis of which regions were lagging the furthest behind in terms of PHC-relevant service coverage indicators from State Statistical Office [15, 16]. Finally, to capture the heterogeneity of the population, the selection of regions considered the education levels and occupations of the citizens, the percentage of citizens aged over 65 years, and spatial distribution of different ethnicities in the country.

The assessment had an integrated focus on rural areas. Based on the latest census data from the State Statistical Office [17], almost 4 out of 10 inhabitants of North Macedonia (38.4%) live in rural areas. According to the Law on the territorial organization of local self-government [18], rural settlements (villages) are defined as monofunctional populated areas, in which one business activity is prevalent and the area has both agricultural features and function. Urban settlements are defined as residential areas with more than 3,000 inhabitants, with a developed structure of various economic activities and with more than 51% of the employees working in the secondary and tertiary sectors [18]. Small urban settlements are defined as having fewer than 20,000 inhabitants [18].

### Computer Assisted Telephone Interview (CATI) Survey

During the period 19–28 November 2022, a WHO-commissioned nationally representative public opinion CATI survey (framed in accordance with the Tanahashi domains in Table 1) was conducted by the TIM Institute. It covered with 1139 respondents (adult citizens of North Macedonia), with an estimated margin of error of + 2.95 percentage points at the 95% level of confidence (confidence interval 95%). A multi-stage stratified sample was used to reflect the demographic characteristics of the population. The sample was distributed proportionally in urban and rural areas in all eight statistical regions of the country. The questionnaire was administered in Macedonian language for ethnic Macedonians and members of non-majority communities, and in Albanian language for ethnic Albanians. Additional language versions were not considered as people with other ethnic affiliations in the country generally speak either Macedonian or Albanian. All data was anonymized and personal identifying information removed, and safe data storage practices were followed. For analyzing the data, the following statistical methods were used: chi-square, t-test and bivariate correlation (Spearman, Kendall’s tau). The survey allowed for a comparison of rural and urban settings, and across other equity stratifiers. Stratification was done by region of the country, socioeconomic status, education, ethnicity, sex, age, persons having a disability, and persons having one or more chronic conditions. The survey instrument can be accessed in the report [19] being published in parallel to this article.

### Key Informant Interviews

The study included a total of 24 in-depth interviews: 12 with relevant national stakeholders and 12 with health professionals from rural and small urban settlements in the four selected regions (3 in each region, constituting a general practitioner/family doctor, nurse, and director of health centre from a rural and/or small urban settlement). Data collection was conducted between November 2022 and January 2023 via semi-structured interviews by the TIM Institute’s team of experienced qualitative researchers familiar with the national context. Interviews were done in Albanian language for ethnic Albanians, and in Macedonian for all others. All interviews were audio recorded, transcribed verbatim, and coded and triangulated as described. Sampling of key informants was designed to include a range of relevant national stakeholders, such as representatives from the Ministry of Health, Health Insurance Fund, Institute for Public Health, Ministry of Labour and Social Policy, international organizations, professional associations and civil society organizations; and health professionals from rural and small urban settlements.

### Focus Group Discussions

In December 2022, focus groups were conducted in the four regions, with adults from subpopulations in situations of vulnerability in rural areas and small urban settlements. The participants in the focus groups were citizens of North Macedonia aged 18 + years living in one of these regions. A total of 12 focus group discussions were held, allowing three in each region that covered different age categories: 18–34 years, 35–64 years, and people aged 65 + years. All focus groups had an equal female-to-male ratio. The group composition strived to match the ethnicities of the populations living in the regions (e.g., Polog region had two groups of Albanians and one of Macedonians, whereas in the East region, there were two groups of Macedonians and one of Roma individuals).

Each focus group consisted of 8–10 participants. The following quotas were applied when recruiting participants: at least 2 participants in each region did not have health insurance; at least 25% of participants in each group had completed secondary school education; no participant in the focus group had higher than the average monthly income (31,407 Macedonian denar [20]) which is 537 USD using October 2023 exchange rates), and 25% had no personal income. One additional focus group comprised individuals with disabilities, with participants from all four selected regions.

Each focus group discussion lasted around 90 min. A guide was used to ensure a semi-structured discussions among participants. Each discussion was organized into two parts: in the first part, for a duration of 70 min, all participants discussed barriers and facilitating factors in relation to health services; and in the second part, for 20 min, the group discussion proceeded only with women participants talking about women’s health and women’s specific gender-related issues impacting on access to health services. Discussions were held in Macedonian and Albanian languages depending on the ethnicity of the participants.

### Desk/literature review

Done by the TIM Institute, the rapid review considered relevant publications produced during 2012–2022. The electronic search strategy was first implemented via PubMed, Embase and Google Scholar, in English, which identified very few articles for North Macedonia despite efforts to widen the search terms. The grey literature came from stakeholder websites in North Macedonia, including: (i) public institutions, such as the IPH and the Health Insurance Fund of the Republic of North Macedonia (HIF), the Ministry of Health, and the Commission for Prevention and Protection against Discrimination; (ii) Civil Society Organizations (including those working with marginalized population groups); (iii) targeted sources, such as international treaty bodies; (iv) international databases, such as the WHO Health for All database, Eurostat, WHO Global Health Observatory, and the World Bank datasets; and (v) legislation in North Macedonia, principally through the collection amassed on the Akademika.mk website.

### Data triangulation and analysis

**Fig. 2:**
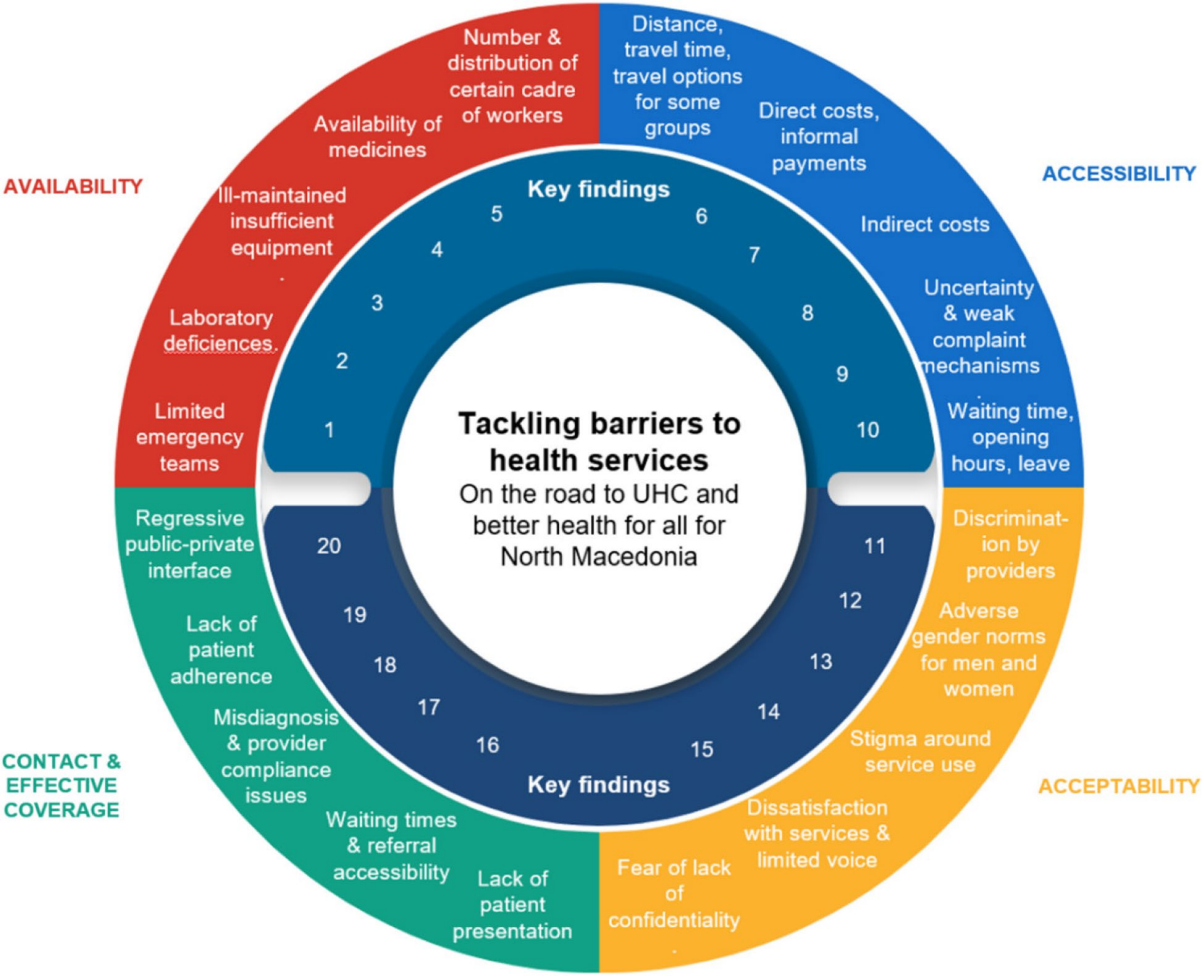
Overview of key findings. Source: WHO Regional Office for Europe (forthcoming)

All data emerging from these distinct sources were processed separately and initially analyzed in accordance with the Tanahashi domains. The quantitative data was analyzed in SPSS and the tables transferred to Excel. The literature review report of findings, all focus group discussion transcripts, and all key informant interview transcripts were done in Word and then coded in Nvivo. Coding of both the quantitative and qualitative findings drew on a detailed guide developed by WHO for coding, based on the Tanahashi framework (see Table 1). The data coding and analysis used both inductive methods and deductive approaches; that is, the Tanahashi framework guided the analysis but both Tim Institute and WHO watched for emerging themes from the data itself. Joint analysis sessions were conducted between TIM Institute and WHO, where data for different variables were interpreted and stratification explored, and triangulation advanced. WHO took a lead in developing the overview of emerging key findings from the study (Fig. 2) based on a cross-analysis and grouping of key emerging themes for each Tanahashi domain. This later became the basis for reporting on the data, and the basis for soliciting stakeholder feedback through the participatory workshop in March 2023.

### Participatory approaches

In addition to the ongoing involvement of the oversight committee with members from the Ministry of Health and Health Insurance Fund, participatory platforms were convened on two occasions. The first was to inform the study protocol. Following WHO orientations on the methods and preliminary adaptations of the approach for the country context in April–May 2022, on 6 June 2022, an inception meeting with 25 + national stakeholders was convened online and in-person to review the draft study plans and give feedback/inputs. A second meeting of national health stakeholders was convened on 13–15 March 2023 in Strumica, North Macedonia, to review the emerging findings and contextualize them in policy and programming opportunities for action. It was jointly organized by the Ministry of Health, WHO and the European Union (EU) Delegation in North Macedonia. WHO and TIM Institute used the overview of emerging key findings (Fig. 2, organized per the Tanahashi domains) to inform the Strumica meeting design.

## Results

An overview of key findings is featured in Fig. 2, followed by a non-exhaustive description of select findings only. The study’s full findings can be accessed in the report [19] being published in parallel to this article. This section also describes the emerging ways forward identified through participatory deliberations.

### Availability

#### Availability of health workers

Evidence emerged from across the study’s four sources of an insufficient number of health workers, in general across the country, but particularly in more disadvantaged regions and rural areas and for certain types of health professionals. For the latest year for which data were available (2019), the number of doctors per 100 000 population was approaching – yet still below – the European Union (EU) average [1].

The number of doctors and other health professionals in rural areas is not proportionate to the portion of the population which lives in them (38.4%) [17]. According to the Institute for Public Health (IPH), in 2018 the total number of doctors was 1470, and 21% (or 308) of them worked in rural areas [21]. Likewise, only 20% of other health personnel worked in rural areas [21].

Regarding perception of health workforce sufficiency, the CATI survey found that only 6 out of 10 respondents reported sufficient numbers of General Practitioners (GPs)/family doctors and dentists in their communities, dropping to only 3 out of 10 reporting sufficient numbers of gynaecologists and paediatricians. There were significant rural–urban differences, with rural respondents being 3 or more times as likely—varying as per the type of health professional– to report a lack of sufficiency.

Inequity (both interregional and rural–urban) in availability of health workers was a point consistently raised in the key informant interviews. Contributing factors were cited as an outdated map of the health network and outdated criteria for inclusion in it; lack of a strategic approach to attracting, recruiting and retaining health workers in areas outside of Skopje; and administrative and financial hurdles for young doctors to establish their practices.

*Everyone wants to come to Skopje. There is available employment in health centres in other regions, but no one applies for those jobs. Even the children of the managers of the health centres who have a diploma in medicine don’t want to. They want to work in Skopje.* [Subnational Key Informant]

*I will repeat this once again, there is an inadequate territory distribution of health services. Many villages don’t have a registered doctor; these are only in the bigger ones. There are not GPs enough in the small towns, as well.* [National Key Informant]

*Young doctors have to start from scratch. The Fund gives them MKD 40 000 [684 USD using October 2023 exchange rates], which is unsustainable for all the fees they have. Another barrier is that the process of opening a practice is very long, from 8 to 10 months... they don’t have income, and they have to operate.* [National Key Informant]

#### Availability of medicines and health products

Data from across the four study sources revealed an insufficient number of medicines, with qualitative sources pointing to the lack of an updated *Positive list of medicines *[22], inadequate distribution/supply chains, tender processes resulting in severe shortages of certain drugs, and medications for some conditions being less available. Similarly, some medical products and devices were not adequately available. Data from the CATI survey showed that 42% reported using medications for a certain health condition prescribed by a health professional. Of this group, 26% stated these medications were not easily available near where they lived. Focus group participants with disabilities reported that the medical aids (crutches, walking sticks, wheelchairs, hearing aids) they received through the HIF were outdated, of lower quality and lesser durability, prompting them to pay out of pocket for modern medical devices and aids.

The literature review found an underprovision (below EU levels) of modern medical technologies for diagnostic purposes and uneven spatial distribution of certain equipment [3, 23]. Focus group discussions and key informant interviews highlighted serious barriers in terms of old and malfunctioning equipment, on one hand, and a lack of training and appropriately skilled medical staff to use the new equipment, on the other hand. Furthermore, these sources indicated that continuous maintenance of the equipment was not being provided.

*It’s not just equipment that’s missing. It is necessary to maintain it, and to have staff who will be trained and know how to properly handle the device and read the results.* [National Key Informant]

#### Availability of laboratory and emergency services

Focus group discussions and key informant interviews reported that the availability of laboratory services in rural areas increased by some GPs contracting with nearby laboratories. Adding more layered information, qualitative sources also reported that challenges persist due to those services only being available at certain times. The reported limited availability of a range of biochemical tests and lack of biochemical specialists in some small urban settlements and rural areas hindered accreditation of laboratories for specific diagnostic analysis.

*Only one day, on Tuesday samples are given for analysis, and on the other days patients will have to be sent to the nearest laboratory, if something is urgent: either in Bogdanci or Valandovo. We have to travel 20 or 30 kilometres. [Male focus group participant, village of Pirava, Southeast region]*

In 2021 there were 284 emergency medical teams (EMTs) nationwide, falling short of 308 EMTs prescribed by legislation [24]. When considering only the ratio per population, geographical differences were found in the availability of EMTs in various health centres across the country, particularly impacting major cities, including the capital Skopje, along with Kumanovo and Tetovo [24]. Building on the literature review, focus group discussions suggested that geographical terrain, distances between villages, population ageing, car ownership linked to socioeconomic status – among other factors – can also play a role in defining whether the prescribed number of EMTs (including ambulances) is enough for a given area.

*We are in great need of an ambulance because the municipality of Krivogastani covers many villages with old people, and everyone needs an ambulance. We can call, but even if they come, it’s from Prilep or from Krushevo, and it is far away. [Female focus group participant, village of Krivogastani, Pelagonia]*

*Even if he is not a dead man, he will die before they arrive. [Male focus group participant, Vinica, East region]*

### Accessibility

#### Geographic, time-related and transport accessibility

The CATI survey showed that, while high numbers of people felt that they were (very or somewhat) close to pharmacies and family doctors, fewer felt that they were close to laboratories, health centres, dentists, gynaecologists and specialist centres. There were clear geographical inequities, with respondents living in rural areas reporting greater distance from services. For instance: over 70% of respondents in rural areas reported being distant from the nearest health centre, compared to 21% in urban areas, and 78% of respondents in rural areas reported being distant from specialists, compared to 40% in urban areas.

Of all respondents living in rural settlements, one third (33.2%) indicated that transportation time was a barrier to reaching a hospital while 41.1% stated it was a barrier to reaching a specialist. The CATI survey as well as the focus group findings showed that transportation time was more frequently reported as a barrier to reaching health care facilities by older respondents (aged 55 + years), respondents with low education levels and low standard of living, respondents with disabilities, those with chronic health conditions, as well as by farmers and housewives.

*There is no public transport and that is the major problem here. There used to be regular bus lines to the rural areas, to the villages – I haven’t seen anything like that lately. People have to use their own cars or taxis, which is expensive. We are talking about the older population in general.* [Subnational Key Informant]

*After my operation, I had to go on foot from Trizla to the hospital to have the surgical dressing changed. That’s a big problem since I had to walk for 2–3 kilometres. It’s too expensive to go by taxi and we don’t have public transport. [Male focus group participant, Trizla, Pelagonia]*

#### Financial accessibility

A large majority, 96.0% of all CATI survey respondents, reported having health insurance from the Health Insurance Fund (HIF). According to the survey, people less likely to have health insurance from the HIF included: farmers, unemployed people, citizens who have private health insurance, and ethnic Albanians.

The study sources converged to show that, while many services are free-of-charge at point of use (in public services), there can be out-of-pocket (OOP) payments for certain medicines, diagnostic tests, and dentistry and gynaecological services, or requests for informal payments. The desk review highlighted that nearly 7% of households were impoverished, further impoverished or at risk of impoverishment after out-of-pocket payments in 2018, and 6.5% of households experienced catastrophic out-of-pocket payments in 2018 [2]. The CATI survey showed that one third of the respondents (32.9%) reported having serious problems accessing health care due to the cost of health services.

In addition, the CATI survey found that four out of 10 respondents (41.3%) reported informal payments as a serious barrier to accessing health care. This was echoed in the focus groups, with informal payments being “necessary” in some situations to receive quality care, even in the case of deliveries (of babies). The study found that indirect costs for transportation and accommodation, along with missed work, also posed barriers. According to the CATI survey, four out of 10 respondents (40.5%) reported having a serious problem when accessing health care due to indirect costs. Of all respondents living in rural areas and small urban settlements, 49.8% and 43.7%, respectively, indicated that the indirect costs were serious barriers to accessing health care, compared to 26.7% of respondents living in Skopje.

Echoing desk review findings that out-of-pocket payments for outpatient medicines are a major driver of catastrophic spending [2], the CATI survey found that nearly half of all respondents (45.1%) reported the cost of medicines and health products as being a serious problem when accessing health care. The cost of medicines and health products is a serious problem for people living on the margins of poverty. Most of the respondents (81.8%) who felt they “barely make ends meet” and use medications for their health condition reported having serious problems accessing health services due to the cost of medicines. In addition, older people, people living in rural areas and people with disabilities were also disproportionately more likely to indicate that the cost of medicines and health products was a serious problem when accessing health care.

*The highest expenses are related to therapy; this is the main problem. We know that 80% of the medication isn’t covered by the Fund. Additionally, there are several types of medication that are covered by the Fund, for hypertension, diabetes, obesity, and the biggest barrier is that they can’t be prescribed by the doctor covered by the HIF; [the patients] have to go to a specialist. Since some of them can’t go, they don’t have time, there are no doctor’s appointments, etc., some of them are forced to buy the medication at their own expense.* [Subnational Key Informant]

#### Organizational barriers

The CATI survey found that waiting time for an appointment was reported by nearly six out of every 10 respondents (58%) as a serious problem when accessing health care, posing by far the most significant organizational barrier. Those who reported being affected were more likely to be older people, employed individuals, and people with chronic conditions. The focus group findings suggested that an additional factor was the waiting *at* the facility for the appointment itself.

*“It’s easier when you go to your doctor covered by the HIF, but everything after that is a problem. You go to see a specialist with an appointment and you don’t know when you go home. If you have an appointment at 9 o’clock in the morning, you will have your check-up at 3 o’clock in the afternoon.” [Male focus group participant, village of Pirava, Southeast region]*

### Acceptability

#### Gender norms, roles and relations

One quarter of the CATI survey respondents believed that “many” or “some” women could have problems getting permission from other family members to access treatment. More women (30%) than men (21%) were likely to indicate that this was an issue. Among female respondents and considering occupations, 51% of housewives believed that women have such permission-related problems, compared to 20% of women employed outside of the home. Women living in rural areas were much more likely than women in urban areas to share this opinion (41% compared to 24%, respectively). Important ethnic differences emerged: 53.3% of Roma women and 48.3% of Albanian women, compared with 23.2% of Macedonian women believed that women have problems getting permission from other family members to access treatment. Focus group discussions as well as key informants provided additional insights into the gender norms that inhibit access to health care among the Roma community, highlighting stigmatization associated with unmarried women or girls requiring gynaecological services, along with the importance of seeing female health care providers.

*Some young women can visit a doctor, but that has to be hidden. There is a certain belief here – she is a young woman who is not married, she mustn’t go to a gynaecologist. [Female focus group participant, Vinica, East region]*

Data from the CATI survey showed that three out of 10 respondents believed that many or some men had problems accessing health care when sick because it was expected for men not to seek help. Having this perception was considerably more pronounced among ethnic Roma (63.3%) and ethnic Albanians (43.6%), compared to 23.0% of ethnic Macedonians. Similarly, this perception was more prevalent among people with no education (61.5%), compared to 23.2% of respondents with higher education, and among rural (35%) compared to urban (26%) respondents.

#### Discrimination on different grounds

CATI study respondents were asked whether they had ever felt discriminated against by a health care worker based on their ethnicity, place of residence, religion, education level, health status, sex/gender, age, occupation, and/or marital status. Around 6% of the respondents felt discriminated against based on their ethnicity, and less than 5% of the respondents felt discriminated against by a health care worker based on other grounds. That said, specific population subgroups were more likely to report experiencing discrimination compared to others. For example, 47% of Roma respondents felt discriminated by a health care worker based on their ethnicity, as did 15% of respondents with no education, based on their education level and 19% of farmers based on their occupation.

Focus group discussions exposed discrimination based on socio-economic status, describing how citizens with higher socio-economic status receive different and better-quality treatment compared to people with lower socioeconomic status, and to people living in rural and remote areas.

*If someone from high society comes to the doctor, he will have a special treatment, I have a different treatment, and someone who comes from a mountain village will have a completely different treatment. So, everything is according to the clothes, according to the status…in our country, this is normal. [Male focus group participant with a disability, Polog region]*

#### Ability to ask questions, privacy and confidentiality

To explore perceptions of quality of health services (including patient centeredness), respondents in this study were asked whether they felt free to ask questions and/or share doubts with health providers. Seven out of 10 respondents (71%) reported that they always felt free to ask questions or share doubts, leaving almost three in 10 (29% of respondents) feeling that they could not (responding never or sometimes). Respondents who reported they “barely make ends meet” were more than twice as likely to respond that they did not feel free to ask questions and/or share doubts with health providers compared to respondents who felt that they “live very well” (37.1% compared to 15.4%, respectively). Ethnic Roma and people with disabilities were also more likely to report this.

According to the CATI survey, a lack of privacy and confidentiality was reported to be an issue for accessing health care by 38.8% of respondents. This sentiment was reported more frequently among Roma (57%) and ethnic Albanians (45%) compared to ethnic Macedonians (36.1%). It was also more likely among respondents living in small urban settlements (49,4%) and rural areas (39,7%), compared to those living in Skopje (24,1%), and among women compared to men (40,9% compared to 36,6%, respectively).

### Contact and effective coverage

In addition to services and medical products being available, accessible, and acceptable, effective coverage is enabled through diagnostic accuracy, effective referrals (and back referrals), treatment adherence, and provider compliance, among other factors that facilitate quality.

#### Perceptions of misdiagnosis

The CATI survey indicated that 10.4% of respondents reported being misdiagnosed by a health care worker, while 86% stated they had never been mis-diagnosed. The key informant interviews and focus group discussions revealed contributing factors, including potential lack of resources for diagnostic accuracy (such as lack of or outdated diagnostic equipment), limited scope of practice of primary care doctors, and/or high referral rate to specialists.

*If the family doctor discovers that the patient has a problem with the prostate, he does not have the right to give him a blood and urine test for the prostate. He has to send him to a specialist, and there are no appointments. The specialist, on the other hand, will send him to do a laboratory and who knows how long he will wait if there are no reagents. So that's how complicated things get. If the family doctor was given more authority to prepare the patient before sending him to the specialist, it would be simpler. *[National Key Informant]

#### Lack of specialist appointments

The CATI survey revealed that more than half of respondents had serious problems accessing health care due to lack of available specialist appointments (57.2%). Some groups of respondents were found to be more likely to report such a shortage of specialist appointments. These included respondents with chronic conditions, compared to those without (71.2% and 50.1% respectively); and respondents who reported “barely making ends meet”, compared to those “living very well” (68.5% and 46.2%, respectively).

*I have a chronic disease that requires regular control and examinations such as computed tomography and magnetic resonance. It is very difficult to make an appointment. I can wait for 3 months and not be able to find an appointment. [Male focus group participant, Krusevo, Pelagonia Region]*

The key informant interviews and focus group discussions highlighted the lack of specialist appointments available on the information system *Moj Termin* (Moj Tepмин (mojtermin.mk) as being a key problem, driven by lack of specialists, their centralization in Skopje, and an unsuccessful notification system for identifying available appointments. Study participants reported that problems accessing timely services in public secondary or tertiary health care was leading to the outflow of patients to the private health care sector (with enhanced risk of financial hardship), as well as people using connections/networks or bribing doctors, reaching for alternative treatment methods or even completely terminating their treatment.

*My father had a heart attack and the specialist here made an urgent appointment in Ohrid for angiograhy for the end of March, and it was October. We collected money from relatives so that we could go to a private clinic so that he could undergo coronarography sooner. It turned out he needed three stents. He had the surgery in a private clinic and we paid for it. Thank God he didn’t wait for it until March and we did everything much earlier. [Female focus group participant, Krushevo, Pelagonia]*

*A patient either goes to a private clinic or waits to die, there is no other choice. Only these two things. [Male focus group participant, Furka, South-East Region]*

### Emerging ways forward to address the barriers

As noted in the methods section, a key component of the assessment was participatory interpretation of the results and deliberation of the ways forward. For aiding in interpretation and the consideration of action-oriented implications, supplementary frameworks were brought into the process. These included the operational framework for PHC [25] and the WHO Priority Public Health Conditions framework for equity analysis [26], the latter of which is central in WHO work on social determinants of health.

The core emerging action areas – identified as potential means of tackling the barriers and optimizing the facilitating factors – from the national stakeholders meeting held in Strumica on 13–15 March 2023 included but were not limited to: (1) equity-oriented, gender-responsive and rights-based biennial action plans to deliver on the commitments in the National Health Strategy 2021–2030; (2) creation of a plan for piloting – including in rural and small urban settlements – adjustments to overcome key barriers in the context of the PHC reform (e.g., such as the expansion of the competencies/tasks delivered by the primary care workforce); (3) convening of the expert group to oversee the updating of the positive list of medicines and exploration of other potential financial protection adjustments such as introducing an annual cap on payment for medicines and/or exempting more low-income people from co-payments on medicine; (4) work towards a national human resources for health strategy for North Macedonia that addresses challenges including the inequitable geographic distribution of the health workforce; and (5) integrating whole-of-society/whole of government approaches to NCDs in the context of the National Development Strategy in North Macedonia.

While it was generally acknowledged by Strumica workshop participants that measures to close coverage gaps and reduce exposure to risk factors for ill-health will require increased financing, it was concurrently acknowledged that these improvements could bring economic and labour market benefits. This is in line with the findings from the 2020 report by the Lionello, Dimkovski, & Jagrič on the impact of the health sector on the national economy in North Macedonia [27].

For example, advancing on the workshop recommendation to strengthen mechanisms to ensure the attraction, recruitment and retention of the health workforce in rural and remote areas was considered a means of providing valuable labour market opportunities for young people in “inner” North Macedonia. This, in turn, could contribute to localized sustainable development and help curb outmigration of the youth themselves and others from rural areas.

Another example was linked to the hiring of administrative staff to support practices at primary care levels. Workshop participants discussed the burden posed to primary care doctors and nurses by all administrative work for the practice, to which they linked some of the study’s identified barriers. Participants reported that the burden takes away from health workers’ capacity to do curative as well as promotive and preventive work (e.g., home visits and multi-stakeholder/intersectoral work for the WHO Best Buys for NCDs [28]). It also limits their ability to provide integrated health and social care for ageing populations, persons with disabilities and others experiencing vulnerability. By hiring administrative staff for all primary care practices, the health sector would not only enhance equitable service delivery, efficiency and effectiveness, but generate 1,500 new employment opportunities in the country.

## Discussion

This advanced convergent parallel mixed methods study explored barriers and facilitating factors to effective coverage with health services as experienced by adult citizens in North Macedonia, with a particular focus on those experienced by subpopulations living in rural areas and small urban settlements and in situations of vulnerability. The study was grounded in the Tanahashi framework for effective coverage and drew on additional frameworks for both granular interpretation and bridging the research-to-policymaking/practice divide.

### Summary, interpretation, and use

The key barriers identified through the study included but were not limited to the following for rural areas and populations experiencing vulnerability: an inadequate number of health workers, in general and in particular in the more disadvantaged regions of the country; insufficient number of outpatient medicines on the Positive List of Medicines; distance and transportation obstacles, including indirect travel costs, particularly in rural areas; adverse gender norms for both women and men; perceived discrimination by providers on multiple grounds; bottlenecks to getting appointments for referrals including waiting times, particularly impacting older people, employed individuals, and people with chronic conditions; and lack of patient adherence, due to costs of medicines and health products, among other factors.

The study findings have relevance in the context of the current Primary Health Care reform in North Macedonia. They can be interpreted in relation to key areas for PHC-oriented health systems strengthening: health workforce, service delivery modalities, health financing, and digital health, amongst others. This was done participatively through the Strumica workshop, as described above. The detailed summary from that workshop can be found in the annex of the full report of findings being published in parallel to this article [19].

At the time of writing, members of the barriers assessment oversight committee from the Ministry of Health and Health Insurance Fund reported drawing on barriers assessment findings for decision-making and planning. Study findings have fed into decision-making regarding: enhancing the competences of health professionals at primary care levels; strengthening laboratory services in rural and remote areas; updating the Positive Drug List, advancing work towards a national health workforce strategy with an embedded focus on workforce issues in rural and remote areas; hiring administrative staff to work as a key part of primary practices so as to not overburden doctors and nurses with administrative tasks, and; considering ways to potentially adjust strategic purchasing to influence quality and equity in health.

### Benefits of and lessons from the mixed methods approach

The assessment used mixed methods to enhance the overall quality of the study by drawing insights from both quantitative and qualitative data, in line with Creswell and Plano Clark, 2017 [29]. For instance, quantitative data elucidated the extent of population experiencing certain barriers and – where sample sizes permitted – which subpopulations may be particularly impacted, as well as certain interactions between barriers. Qualitative data then deepened the exploration by describing how patient pathways are influenced by a range of dynamic supply and demand-side factors operating in context-specific interfaces between the patient and the services, with barriers often compounding and intersecting. Quantitative and qualitative sources together more comprehensively described the role of health systems and wider key social and environmental determinants in influencing health inequities. There was a lack of such mixed methods research in North Macedonia, and this study aimed to fill the gaps. Participatory approaches also aimed to optimize the tailoring of the research instruments to the national context, build on existing relevant work, and bridge the research-to-policymaking/practice divide.

The draft WHO handbook for assessing barriers to effective coverage with health services did not include a quantitative data collection instrument, only guidance for the exploration of existing data sets, prior to its piloting in North Macedonia. Hence, the development of a survey tool based on the Tanahashi framework by the Tim Institute, with help refining the indicators by WHO, was a significant advancement in the methods.

### Value added in relation to existing literature

The assessment adds value to existing data sources for health systems performance for North Macedonia, including Winkelmann J, et al. (2022) and Dimkovski V, Mosca I (2021) [1, 2]. It adds “demand-side” perspectives, i.e., data on the self-reported experiences of the population in need of and with rights to services. Such data came through the focus group discussions and CATI survey. In addition, this assessment built on existing descriptions of system performance at aggregate levels through its stratification of data to capture health equity considerations. For example, not only did the assessment (through the CATI survey) quantify respondents reporting having serious problems accessing health services due to the cost of medicines; it looked deeper at the extent to which low-income respondents who felt they “barely make ends meet” AND use medications for their health condition feel this way (the resulting figure was 81.8%). This highlights the gravity of this barrier for the poorest populations and those persons with chronic conditions, both of which are priorities in the Primary Health Care reform.

Through the participatory dimension of the study that delved ways to tackle barriers or optimize facilitating factors, the recommendations in papers such as that by Bryar R et al. on developing modern primary care nursing in North Macedonia [30] were validated and further explored through an equity lens. Through its mixed methods approach, the study also added further contextual information to existing studies on health systems performance for specific subpopulations, such as those on older people [31], Roma women [32], and LGBTIQ + persons [33].

### Limitations

The assessment focused predominantly on barriers that occur at the patient-provider interface and, to some extent, facilitating factors in effective coverage with health services. It was not designed to delve in detail on the upstream causes of differential exposure to risk factors for ill-health, which is often driven by factors outside of the health sector, nor did it analyse in-depth the root causes of the reported barriers. The latter is important, for each singular barrier, when refining the steps for the ways forward and for the interventions to succeed. In North Macedonia, further research is opportune on social and environmental determinants of health. Application of a Health-in-All-Policies approach will be critical to improve overall population health and reduce health inequities. In addition, additional ongoing analysis of specific “causes of the causes” of health system bottlenecks will be important to build into ongoing planning, monitoring and evaluation cycles.

The CATI survey provided opinions expressed at a certain point in time. All sample surveys may be subject to multiple sources of error, including – but not limited to – sampling error, coverage error and measurement error. Furthermore, the findings from the focus group discussions and KIIs should be considered as indicative of the participants’ viewpoints and they should not be accepted unconditionally as representative of the attitudes and the opinions of the whole population or all relevant stakeholders in North Macedonia.

Finally, this study focused on barriers to effective coverage with health services experienced by adults in North Macedonia, and was not designed to specifically consider barriers experienced by children. The decision by the study team to limit the sampling framework to focus on adults was done based on a pragmatic calculation of resources available for the study and a focus on reinforcing/strengthening NCDs services in the Primary Health Care reform. The study team is aware that children experience important barriers to NCD-related and other health services, and encourages further research in this area. Likewise, the sampling framework focused on citizens of North Macedonia. Other resources [12, 34, 35] should be referred to and potential follow-up research carried out to better understand the situation of migrants and persons with refugee status in the country.

## Conclusions

Through its focus on barriers that occur at the patient-provider interface, the assessment produced evidence for the ongoing Primary Health Care reform in North Macedonia and related health system strengthening processes. The assessment responded to the lack of comprehensive data and research in North Macedonia on barriers to effective coverage with health services, across the dimensions of the Tanahashi framework, with a particular focus on socio-spatial differences and subpopulations in situations of vulnerability. By doing so, it shed further light on causes of stagnation/bottlenecks in progress towards UHC, as well as the need for systems thinking in finding solutions and identifying ways forward.

Mixed methods enabled exploration of the extent of barriers and facilitating factors, while also giving explanations on how and why such barriers emerge and interact/compound, and how they impact certain subpopulations more than others. This assessment was a pilot of a draft WHO draft handbook for conducting assessments of barriers to effective coverage with health services (WHO, forthcoming). The assessment provided important lessons learnt and additional orientations for the handbook, in particular through the application of the CATI survey methods advanced by the TIM institute in North Macedonia.

## Data Availability

The data that support the findings of this study are available from the WHO Country Office for North Macedonia but restrictions apply to the availability of these data, which were used under license for the current study, and so are not publicly available. Data [with all personal identification and otherwise identifying information removed] are however available from the authors upon reasonable request and with permission of WHO.

## Abbreviations

CSO: Civil Society Organization
CATI: Computer Assisted Telephone Interview
EU: European Union
ESE: Association for Emancipation, Solidarity and Equality of Women
GPs: General Practitioners
HERA: Association for Health Education and Research
HIF: Health Insurance Fund
IPH: Institute for Public Health
MKD: Macedonian Denar
NDS: National Development Strategy
NGO: Nongovernmental Organization
OOP: Out-of-pocket expenditures
PHC: Primary Health Care
UHC: Universal Health Coverage
UNICEF: United Nations’ Children Fund
WHO: World Health Organization
